# Laser-Plasma Driven Synthesis of Carbon-Based Nanomaterials

**DOI:** 10.1038/s41598-017-12243-4

**Published:** 2017-09-20

**Authors:** M. Barberio, P. Antici

**Affiliations:** INRS-EMT, 1650 Boul. Lionel Boulet, J3X1S2 Varennes, Canada

## Abstract

In this paper we introduce a laser-plasma driven method for the production of carbon based nanomaterials and in particular bi- and few-layers of Graphene. This is obtained by using laser-plasma exfoliation of amorphous Graphite in a liquid solution, employing a laser with energy in the order of 0.5 J/mm^2^. Raman and XPS analysis of a carbon colloidal performed at different irradiation stages indicate the formation of Graphene multilayers with an increasing number of layers: the amount of layers varies from a monolayer obtained in the first few seconds of the laser irradiation, up to two layers obtained after 10 s, and finally to Graphite and amorphous carbon obtained after 40 s of irradiation. The obtained colloidals are pure, without any presence of impurities or Graphene oxides, and can easily be deposited onto large surfaces (in the order of cm^2^) for being characterized or for being used in diverse applications.

## Introduction

Graphene is one of the most promising materials for nanoscience applications^1–3^. This simple 2-D material, composed of a single layer of carbon atoms, is characterized by high electron mobility and a field-generated band gap, therefore, it is defined as a zero-gap semiconductor^4^. Nevertheless, technological applications that take advantage of Graphene electronic transport properties require structurally coherent Graphene on a large scale (e.g. wafer scale), or large arrays of Graphene flakes positioned with a unique azimuthal orientation on a substrate.

In the last decades, many preparation processes have been suggested in order to improve the quality of Graphene and its properties. The first method was the mechanical exfoliation from Graphite proposed in 2004^4^, which – being a low-budget technique - strongly contributed to arousing interest for Graphene. However, the produced Graphene flakes have irregular shapes and a random azimuthal orientation. Currently, Chemical Vapor Deposition (CVD) on transition metal substrates^5–7^ can produce the best quality Graphene. In particular, Graphene obtained with CDV deposited on copper foils forms large uniform layers. Nonetheless, the electrical properties of CVD Graphene cannot be tested on a metal substrate, since the testing requires a transfer process of the Graphene layer onto an appropriate insulating substrate. The transfer process often affects the Graphene integrity, its properties, and thus the overall performance: Wrinkle formation, impurities, Graphene tearing, and other structural defects can occur during the transfer. Moreover, CVD Graphene grown on substrates has its size limited by the reactor size, which restricts a large-scale production. Alternative methods for the production of Graphene are epitaxial growth on metal surfaces or graphitization of hexagonal SiC^8,9^. In all these cases, the quality of Graphene is strictly related to the substrate properties such as size, crystallinity, purity, since, similarly to the CVD, these production processes require a transfer.

In addition to these processes, recently also laser-driven methods for the synthesis of nanomaterials were proposed as alternative to chemical techniques^10^. The most employed laser method is the Laser Ablation, which can be performed in vacuum, controlled gaseous atmosphere, or in liquid (Laser Ablation Synthesis in Solution - LASiS). Laser methods allow having a precise control in the dimensions and shapes of the produced nanomaterials, with an *in-situ* tuning of the material properties being possible by simply changing the laser and plasma characteristics. While these methods are efficiently used for the synthesis of nanoparticles and quantum dots^11,12^, the high temperatures and pressures reached on the generated plasma prevent their use in the Graphene synthesis. Higher temperatures can induce the amorphization of the carbon structures leading to a complete loss of the hexagonal lattice.

**Figure 1:**
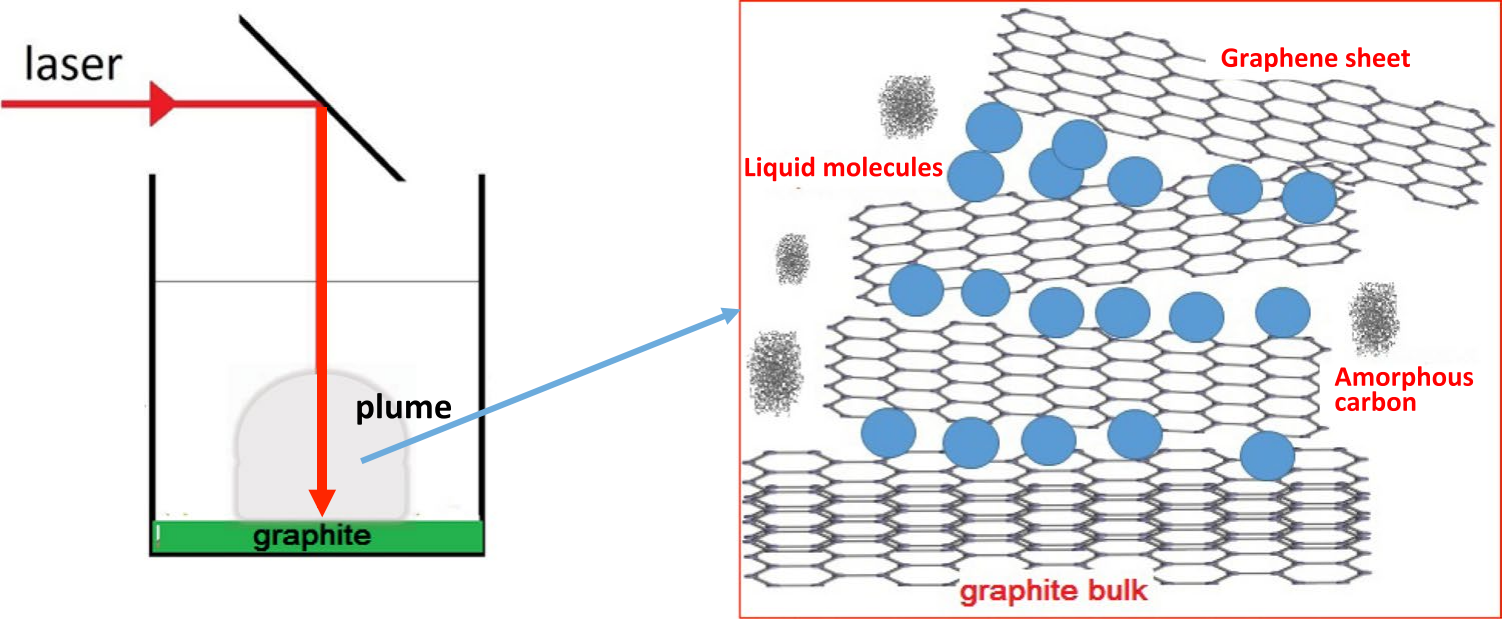
Sketch of the experimental setup and method.

In the following, we propose a simple method for the large-scale production of colloidal solution of Graphene flakes based on the interaction between a higher power laser beam (power of about 100 MW) and a material bulk. The technique (see Fig. 1) is based on the exfoliation of an amorphous Graphite sample located in a liquid solution by using a high power laser irradiation. The laser-carbon interaction in a liquid solution thermalizes the system, preventing to reach the melting temperature. The exfoliated carbon layer is suspended in the solution and can, once collected, easily be deposited (by drop-cast or centrifugation) on a substrate for characterization and application. We synthesize bi-layers of Graphene in 10 s of laser irradiation, demonstrating the possibility of synthesizing mono or multi-layer simply by changing the laser irradiation time.

Our method is similar to other laser-based synthesis processes that have been explored previously (see later), although our synthesis process has the advantage of combining a much easier implementation with a much faster Graphene production, very suitable for large scale production, still enabling high quality optical and electrical properties. In first pioneering works (see refs.^13,14^ the authors suggest forming freestanding 2D nanostructures with a synthesis method based on the Plasma Laser Deposition (PLD), which however is requiring a high vacuum system, a Highly Oriented Pyrolytic Graphite base sample, and a substrate for the Graphene deposition. The method presented in refs.^15,16^ allows the production of Graphene *oxide* at a liquid interface, while in our case we obtain pure Graphene in a colloidal solution. The method proposed in ref.^17^ requires the use of liquid nitrogen and consequently the use of very low temperatures. This, since the nitrogen molecules are crucial for the exfoliation of the graphite. All the above-mentioned techniques are at the base of our proposed method, however, compared to them, we are able to relax some of the required stringent manufacturing characteristics (e.g. low temperature) making our technique more accessible and easier implementable.

## Materials and Methods

### The Laser Exfoliation Method

Our Graphene growth method is based on the laser exfoliation (LE) of amorphous Graphite, realized by using an experimental method that is typically employed in the conventional Laser-method for nanomaterials synthesis, the “Laser Ablation Synthesis in Solution” (LASiS). The preparation of Graphene flakes using LE has the advantage of not requiring vacuum conditions (similarly to CVD or Epitaxial growth), and as such allows achieving layers with higher purity produced in diverse solutions (permitting to choose the solution which is most suitable for the applications). In our experimental setup, a Graphite plate, with nominal purity of 99.99% and dimensions of about 1 cm × 1 cm, is placed on the bottom of a vessel cuvette containing an aqueous solution of acetone (90%). We chose as solvent acetone (oxygen-free ambient) for preventing the oxidation of nanoflakes during the synthesis process and the formation of Graphene oxide. The plate is ablated with the second harmonic (532 nm) of a pulsed YAG:LASER. The laser spot size on the target surface is of about 1 mm^2^. The laser fluence is about 0.5 J/pulse (with a pulselength of 7 ns). The laser source used for the LE reaches the target from the top surface of a cuvette. The colloidal solution, produced at different irradiation times, is transferred to a copper surface by drop-casting and heated in air up to 70 °C for solvent evaporation. Analytical characterization of each deposited drop was performed by morphological (AFM-SEM) and chemical (RAMAN and XPS) information. Differently from the classical Laser Ablation technique employed for the synthesis of nanoparticles, where the approach is typically bottom/up (i.e. atoms nucleate producing nanoparticles), the physical phenomena involved in the LE can be attributed to both, a top/down and a bottom/up approach: When the laser irradiates the carbon surface, it generates (similarly as for the generation of nanoparticles) different phenomena such as the detachment of single carbon atoms (which aggregate in amorphous carbon particles (bottom/up approach)), and the exfoliation of a single or multi-layer of Graphene (top/down approach). All these detached materials are distributed in the plasma plume and are dispersed in the colloidal solution. While the multi-layer of Graphene stays suspended in the colloidal solutions, the carbon atoms aggregate producing amorphous microparticles.

Morphological analysis of the colloidal drops deposited onto a silicon surface indicates the presence of Graphene bi- and multi-layer and of amorphous carbon aggregates generated by the aggregation of single atoms detached during the laser irradiation. Nevertheless, the amount of the Graphene multilayer or the amorphous aggregates changes with irradiation time, indicating that the two phenomena (particle synthesis and Graphene exfoliation) occur at different stages. The presence of amorphous carbon in the solution is higher after the first 10 s of irradiation, clearly indicating that the breaking of the interlayer bonding is the predominant phenomenon in a first phase of the laser irradiation while this effect (the breaking) becomes negligible respect to the amorphous aggregation in the second phase.

The colloidal solution is finally deposited by drop cast on a conductive glass substrate and the film is heated in air to about 50 °C for facilitating the solvent evaporation. The deposited layer is analysed in order to check the possibility of producing very large substrates covered with our produced Graphene multilayers and to test the electrical and optical properties.

### Analytical characterization

AFM images are obtained with the ICON AFM microscope manufactured by the company Bruker working in the tapping mode. Each image is taken with a resolution of 512 × 512 and 1024 × 1024 pixels and a frequency of 1 Hz. Dimensions of the Graphene flakes are computed conducting a statistical analysis on about 100 flakes collected in several AFM images. For each sample, we scan several areas (about 50) in a window of 5 µm × 5 µm (resolution of 1024 × 1024 pixels). SEM analysis was conducted using a field emission SEM microscope (Quanta 200 F FEI/Philips) working with an energy of 20 keV.

XPS measurements are conducted in a UHV chamber equipped for standard surface analysis with a pressure in the range of 10^−9^ torr. Non-monochromatic Mg-Kα X-rays (hν = 1253.64 eV) are used as excitation source. The XPS spectra are calibrated with the C1s peak of a pure carbon sample (energy position located at 284.6 eV). All XPS spectra are corrected for analyzer transmission and the background is subtracted using the straight-line subtraction mode. Moreover, the XPS data are fitted assuming a Gaussian distribution. Finally, the KLL Auger structure of carbon is analyzed in a Derivative mode for evaluating the D parameter. The Raman measurements are taken by a Raman Microscope (Thermo Fisher DXR), equipped with a 532 nm laser and a spot size with a resolution of 1 micron. The spectra are obtained with a 50 × objective (focal length of 15 mm). The spectral resolution, by using the 1800 grooves/mm grating, was estimated to be better than about 2 cm^−1^. Each micro-Raman spectrum was collected in 20 s and three accumulations.

The optical transmittance of the Graphene multilayer are measured by depositing different colloidal quantities onto a conductive glass substrate, and irradiating the film with a halogen lamp under confocal microscope conditions (Olympus 900 by Horiba). The transmitted spectra are taken using a Triax 320 (Horiba–Jobyn–Yvon) spectrometer working in the 300–800 nm range.

The electrical measurements are conducted on some of the films with best uniformity in coverage (deposited onto a conductive glass). The ohmic film resistance (R) is measured using a Keithley DC Current generator Model 2100 with four point probe, while conductivity (σ) is measured using the following formula:

$${\rm{\sigma }}={\rm{L}}/({\rm{R}}.{\rm{A}})$$

where L is the film thickness measured from the SEM images and A the area covered by the four probe detectors (about 1 cm^2^).

## Results and Discussion

Chemical information on the produced carbon nanostructures is obtained studying the evolution of the G and 2D Raman structures of carbon with increasing irradiation time, while the final confirmation of the Graphene formation in the best colloidal solution is obtained by analyzing the D parameter of the KLL Auger line of carbon in the XPS data. Both measurements (G and 2D Raman and D parameter) are indicated in literature as fingerprints of the carbon allotropes.

The effectiveness of the Raman diagnostic for the individuation of Graphene presence on a surface is well known in literature and generally used as a proof of Graphene formation and deposition^18^. The systematic study in Graf *et al*. (ref.^18^ indicates as most relevant parameters for the individuation of Graphene (single or multilayer) the peak position of the 2D band and the ratio between the G and 2D band intensity. In detail, a Graphene monolayer shows a 2D peak position of 2678.8 cm^−1^ and a G/2D ratio around 0.25; for multilayers of Graphene (from 2 up to 5 layers) the 2D peak becomes larger, splits in different substructures, and the energy position shifts of about 19 eV for 2 layers up to about 26 eV for 6 layers. The G/2D ratio varies from 0.4 (2 layers) up to 0.8 (6 layers).

In addition, the confirmation of Graphene formation can also be obtained by studying the changes in the carbon Auger emission. More precisely, Kaciulis *et al*., in ref.^19^ indicate in the D parameter, which calculates the distance between the positive maximum and negative minimum of the KLL line derivative, the key parameter for the identification of carbon allotropes. Their data indicate for D a value of about 15 eV for Graphene, 21 eV for Graphite and 13 eV for Diamond.

**Figure 2:**
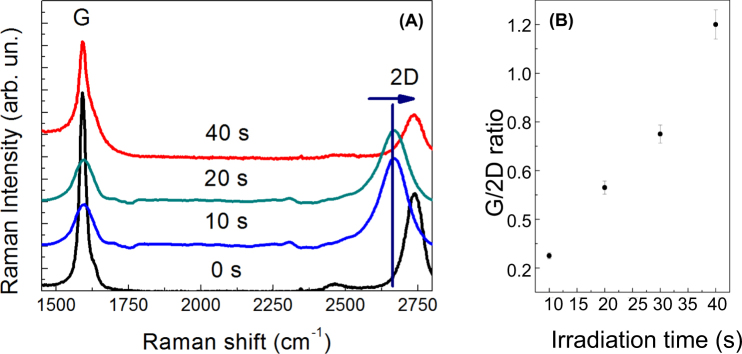
(**A**) Raman spectra of the colloidal solution obtained at different irradiation times and deposited on a copper surface. The black curve is the Raman spectrum of the bulk Graphite taken before irradiation; (**B**) G/2D ratio as function of laser irradiation time.

**Figure 3:**
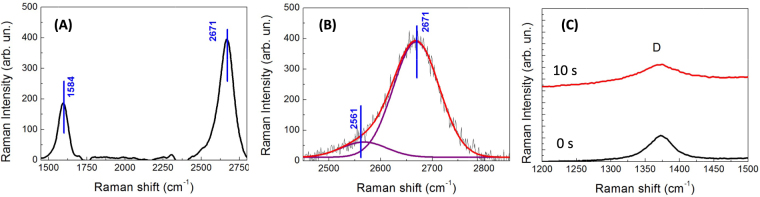
(**A**) Raman spectrum of flakes obtained after 10 s of laser irradiation and deposited on copper; (**B**) details of the 2D structure located around 2650 cm^−1^ (red and violet curves indicate the Gaussian fit); (**C**) details of the (**D**) structures for bulk graphite (black line) and for Graphene bi-layer produced after 10 s of laser irradiation.

**Figure 4:**
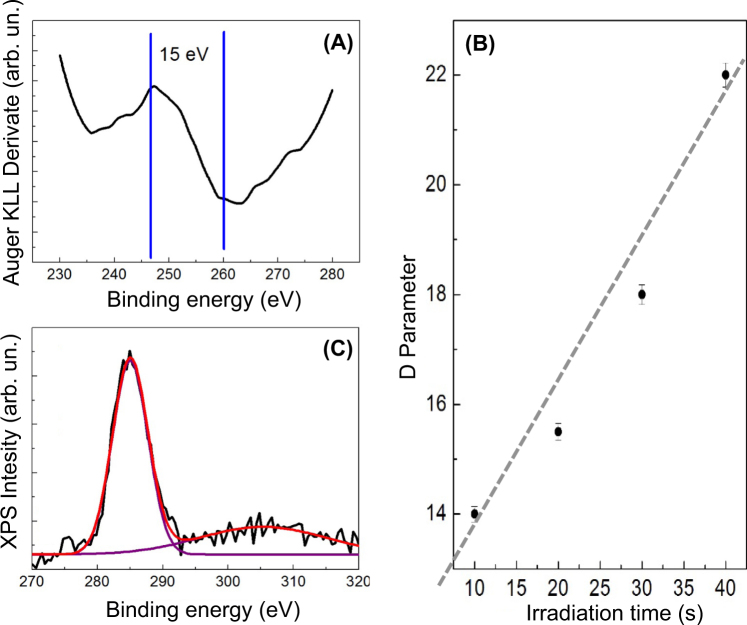
(**A**) KLL Auger spectrum of Grapheme flakes obtained after 10 s of laser irradiation and deposited on copper; (**B**) evolution of the D parameter with irradiation time; (**C**) C1s band of flakes deposited on copper after 10 s of laser irradiation.

In our experiments, we deposited 100 μL of a colloidal solution obtained at different laser irradiation times (10 s, 20 s, 30 s, 40 s, 1 minute) onto a SiO_2_ surface and for each deposited droplet we took a Raman spectrum, following the evolution of the G and 2D bands. As visible from the Raman spectra in Fig. 2A, the 2D energy position and the G/2D ratio strongly change during the laser irradiation. The obtained values for the G/2D ratio (Fig. 2B) range from the typical values of bi-layer Graphene (0.25)^18^ obtained for droplets collected after 10 seconds of irradiation, to Graphite values (>1)^18^ obtained after 30/40 seconds of laser irradiation. A detailed analysis of the Raman spectrum of a colloidal deposited after 10 s of irradiation (Fig. 3A) indicates for the G and 2D energy positions the values of respectively 1584 cm^−1^ and 2671 cm^−1^, which are typical values of 2-layers Graphene. Moreover, the D peak (see the zoom of the Raman spectra displayed in Fig. 3C) related to the defect structures in Graphite (1367 cm^−1^)^15,18^ is strongly reduced after 10 s of irradiation, indicating the formation of states with less lattice imperfections and confirming the formation of Graphene bi-layers. Our assumption is reaffirmed by the fact that the 2D peak (Fig. 3B) is split in 2 structures at 2561 and 2671 cm^−1^, as indicated in Graf *et al*. (ref.^18^) for a bi-layer material. The analysis of the D parameter (Fig. 4), confirms the Raman results, indicating the typical value for Graphene (i.e. a value of 15 eV)^19^ after 10 s of irradiation (see Fig. 4A). The D value increases with irradiation time and reaches the value of 18–19 eV (Graphite value) for 30 s of irradiation (Fig. 4B). Finally the C1s peak (taken during the XPS measurements – see Fig. 4C) shows a single band peak located at an energy of 284.3 eV (characteristic for C-C chemical bonds), without showing the split at 287 eV, characteristic of C-O bonds^15,19^. This indicates the absence of oxygen impurities or Graphene oxide in the colloidal.

Progressive aggregation of nanomaterials in the LASiS plume during the laser irradiation is a well-known phenomenon^10^. The observed starting time for the aggregation (30 s) is identical to what observed for metal and semiconducting nanoparticles^20–22^, indicating a plume expansion and evolution similar to that observed for the synthesis of nanoparticles^11,12^. The main stages in the LASiS exfoliation process can be resumed as follows: the process starts with the absorption of the laser pulse energy by the Graphite target. This generates both the detachment of carbon atoms from the surface and the breaking of the Van Der Waals bonding between the carbon layers in the first micron of the Graphite surface. A plasma plume containing carbon atoms and the exfoliated mono- and bi-layers of carbon expands into the surrounding liquid. During the expansion, the plasma plume cools down and releases energy to the liquid solution. This phenomenon generates a cavitation bubble inside the bulk target, bubble that expands in the liquid and then collapses in a time scale in the order of hundreds of microseconds. The exfoliation of mono- and multilayer and the desorption of carbon atoms are estimated to occur in a timeframe ranging from 10^−6^ and 10^−4^ s after the impact of the laser pulse on the surface (the laser has a pulse duration of about 7 ns). These first phases are followed by a multilayer aggregation and a microparticle nucleation, which both form amorphous carbon structures and Graphite.

**Figure 5:**
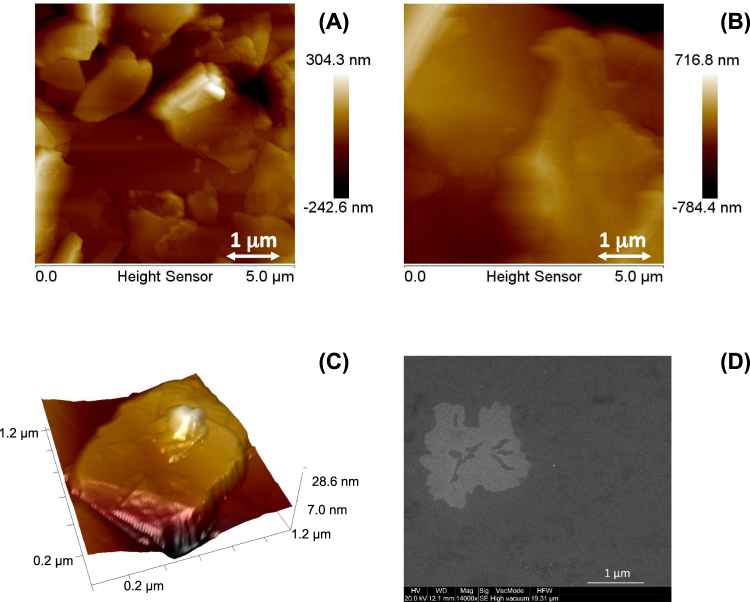
(**A**,**B**) 2D and (**C**) 3D AFM and (**D**) SEM images of the flakes produced after 10s of laser irradiation and deposited on copper.

The final confirmation of Graphene bi-layer formation is obtained by morphological analysis of the small droplet (5 µL) of colloidal deposited onto copper after 10 s of irradiation. The 2D and 3D AFM images and SEM pictures in Fig. 5 clearly indicate the presence of micrometric flakes with dimensions ranging from hundreds of nanometers up to few microns (and having a thickness of 3–4 nanometers) on the copper substrate. This indicates the formation of a Graphene bi-layer (the thickness of Graphene is reported to be in the range of 0.4 to 1.7 nm^23^.

**Figure 6:**
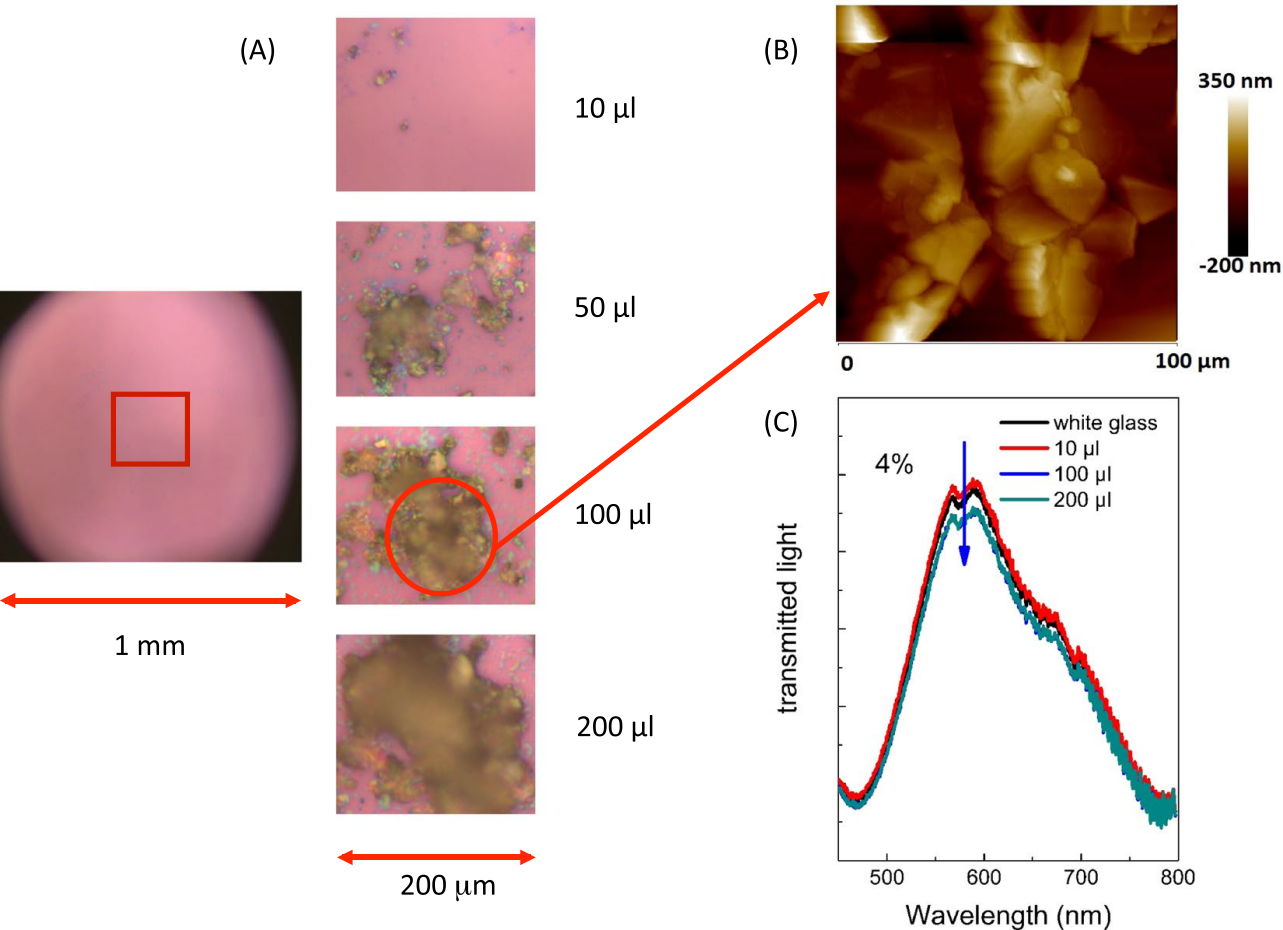
(**A**) Optical images (10x) of Graphene flakes deposited on conductive glass for different amounts of colloidal solution (white glass on the left and on the right a zoom of the deposited films). (**B**) AFM images in the region of best coverage (the limit of the AFM measurements is a window of 100 × 100 µm) and (**C**) optical transmission for films obtained with different colloidal amounts.

**Figure 7:**
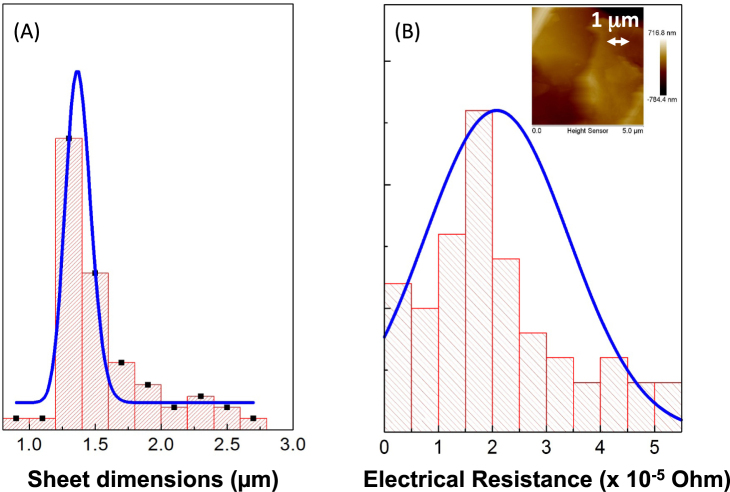
(**A**) Statistical analysis of the flake dimensions (major side). (**B**) Statistical analysis of the film electrical resistance. (Inset) AFM Image of a film section deposited on a conductive substrate for electrical characterizations.

The analysis on colloidals deposited on a glass substrate (Fig. 6) indicated that with the simple drop cast method it is possible to cover very large areas by simply changing the amount of the deposited solution. Figure 6A shows a sequence of substrate coverage (surface area of about 200 µm^2^) obtained with different solution amounts. The area was completely covered with the 200 µL of the colloidal solution. AFM images (Fig. 6B) indicate that the area is fully covered by a uniform film consisting of our Graphene multilayer. Glass optical transmittance (Fig. 6C) was reduced of about 4% at total coverage, indicating that the film is composed only by the Graphene multilayer, without the presence of Graphite aggregates (in this case we would expect a variation in the transmittance of the order of 50% or higher due the high absorbance of amorphous Graphite (order of 90%)). Statistical analysis (see Fig. 7A) conducted on about 500 flakes of Graphene bi-layer deposited on the glass substrate and pictured in AFM images indicates that the flake dimensions are distributed following a LogNormal distribution with an average value of 1.37 μm and a standard deviation of about 6%.

Finally, the electrical measurements conducted on the Graphene film deposited onto a conductive substrate indicate an electrical resistance of 2.08 *10^−5^ Ohm (average value on 50 measurements – see histogram in Fig. 7B) measured on a film with a thickness of 1 µm (see AFM image in the inset in Fig. 7B) and an area of 1 cm^2^. The calculated electrical conductivity is then of about 480 S/m at atmospheric pressure, in agreement with data reported in literature^24^.

## Conclusions

In this paper, we introduce a method to synthesize bi-layers of Graphene using a Laser-Exfoliation based on the Laser Ablation techniques typically employed in the nanoparticle production. The obtained results indicate that the quality and the allotropes of the obtained carbon colloidals are strictly dependent on the laser irradiation time. Graphene bi-layer is obtained in the first 10 s of irradiation while for irradiation time greater than 30 seconds the colloidals are mainly composed by amorphous carbon and Graphite. The obtained bi-layers are characterized by Raman and XPS measurements, which indicate the formation of pure carbon layers with absence of oxygen impurities or Graphene oxide. Moreover the obtained micrometric flakes can be distributed on large areas (order of cm^2^) obtaining uniform films with high optical transmission, indicating that the flakes do not aggregate during deposition. Finally, electric measurements on the deposited films indicate, at atmospheric pressure, a conductivity in the order of 480 S/m, typical for Graphene films.

## References

[1] Avourious P, Dimitrakopoulos C (2012). Graphene: synthesis and applications. Materials Today.

[2] Ferrari AC, Basko DM (2013). Raman spectroscopy as a versatile tool for studying the properties of grapheme. Nature Nanotechnology.

[3] Lee C, Wei X, Kysar JW, Hone J (2008). Measurement of the elastic properties and intrinsic strength of monolayer grapheme. Science.

[4] Novoselov KS (2004). Electric Field Effect in Atomically Thin Carbon Films. Science.

[5] Hagstrom S, Lyon HB, Somorjai GA (1965). Surface Structures on the Clean Platinum (100) Surface. Phys. Rev. Lett..

[6] Zhang Y (2010). Comparison of Graphene Growth on Single-Crystalline and Polycrystalline Ni by Chemical Vapor Deposition. Phys. Chem. Lett..

[7] Yu Q (2008). Graphene segregated on Ni surfaces and transferred to insulators. Appl. Phys. Lett..

[8] Hannon JB, Copel M, Trompet RM (2011). Direct Measurement of the Growth Mode of Graphene on SiC(0001) and SiC(0001). Phys. Rev. Lett..

[9] Araujo PT (2008). Nature of the constant factor in the relation between radial breathing mode frequency and tube diameter for single-wall carbon nanotubes. Phys. Rev. B.

[10] Barberio M, Antici P (2017). *In situ* study of nucleation and aggregation phases for nanoparticles grown by laser-driven methods. Sci. Rep..

[11] Yang GW (2007). Laser ablation in liquids: Applications in the synthesis of nanocrystals. Progress in Materials Science.

[12] Intartaglia R (2013). Laser synthesis of ligand-free bimetallic nanoparticles for plasmonic applications. Phy. Chem. Phys..

[13] Qian M (2011). Formation of graphene sheets through laser exfoliation of highly ordered pyrolytic graphite. Applied Physics Letters.

[14] Kumar P (2013). Laser flash synthesis of graphene and its inorganic analogues: An innovative breakthrough with immense promise. RSC Adv..

[15] Compagnini G (2012). Laser assisted green synthesis of free standing reduced graphene oxides at the water–air interface. Nanotechnology.

[16] Guan YC (2016). Fabrication of Laser-reduced Graphene Oxide in Liquid Nitrogen Environment. Sci. Rep..

[17] Mortazavi SZ, Parvin P, Reyhani A (2012). Fabrication of graphene based on Q-switched Nd:YAG laser ablation of graphite target in liquid nitrogen. Laser Phys. Lett..

[18] Graf D (2008). Spatially Resolved Raman Spectroscopy of Single- and Few-Layer Graphene. Nano Letters.

[19] Kaciulis S, Mezzi A, Calvani P, Trucchi DM (2014). Electron spectroscopy of the main allotropes of carbon. Surf. Interface Anal..

[20] Barberio M, Stranges F, Xu F (2015). Coating geometry of Ag, Ti, Co, Ni, and Al nanoparticles on carbon nanotubes. Applied Surface Science.

[21] Barberio M, Imbrogno A, Stranges F, Bonanno A, Xu F (2015). Controlled synthesis of ZnS quantum dots with cubic crystallinity by laser ablation in solution. Materials Research Express.

[22] Barberio M, Antici P (2015). Nanostructured target fabrication with metal and semiconductor nanoparticles. Materials Research Express.

[23] Shearer CJ, Slattery AD, Stapleton AJ, Shapter JG, Gibson CT (2016). Accurate thickness measurement of grapheme. Nanotechnology.

[24] Rani A, Nam S, Ah OHK, Park M (2010). Electrical Conductivity of Chemically Reduced Graphene Powders under Compression. Carbon Letters.

